# The impact of cue and preparation prompts on attention guidance in goal-directed tasks

**DOI:** 10.3389/fnhum.2024.1397452

**Published:** 2024-07-17

**Authors:** Yahui Li, Yimeng You, Baobao Yu, Yue Lu, Huilin Zhou, Min Tang, Guokun Zuo, Jialin Xu

**Affiliations:** ^1^Cixi Biomedical Research Institute, Wenzhou Medical University, Ningbo, Zhejiang, China; ^2^Ningbo Institute of Materials Technology and Engineering, Chinese Academy of Sciences, Ningbo, Zhejiang, China; ^3^Ningbo Cixi Institute of Biomedical Engineering, Ningbo, Zhejiang, China; ^4^Department of Neurological Rehabilitation, Ningbo Rehabilitation Hospital, Ningbo, Zhejiang, China; ^5^University of Chinese Academy of Sciences, Beijing, China

**Keywords:** attention, visual prompt, event-related potential, brain functional network, graph theoretical analysis

## Abstract

**Introduction:**

In goal-directed tasks, visual prompts before the appearance of goals can make people ready in advance, which helps them to complete the movement better, and the presentation type of the visual prompt is very important. In previous studies, it has not been clear how different types of visual prompts guide attention in goal-directed tasks.

**Methods:**

According to the characteristics of goal-directed tasks, our research designed three different prompts: the cue prompt (featuring static arrow), the preparation prompt (involving dynamic countdown), and the combination prompt of cue and preparation information (simultaneously incorporating arrow and countdown). We used event-related potential components (CNV and P300) and graph theory indicators (clustering coefficient and characteristic path length) under the brain function connection to analyze the attention state of the brain.

**Results:**

The results showed that the combination prompts better guided the participants’ sustained attention during the prompt stage, making them well prepared for the movement. Thus, after the target appeared, the participants had better executive control and achieved a faster response to the target. However, under the combination prompt, the participants consumed more attention resources during the prompt stage.

**Discussion:**

We believe that for the participants with impaired cognitive function, cue prompts or preparation prompts can be considered, which also play a role in guiding the participants’ attention and helping them make motor preparations when less attention resources are consumed. This study provides a neurophysiological and behavioral foundation for the design of visual prompts in goal-directed tasks.

## 1 Introduction

Goal-directed tasks can improve human’s limb motor function by enhancing the neural participation of the brain (Wang et al., 2020). In the task, persons need to prepare for the target to complete the task, and the addition of visual prompt is expected to help them prepare in advance, which is beneficial to their movement (Dean et al., 2012). The goal-directed task with visual prompts consists of two stages: the pre-target stage and the post-target stage. Attention is crucial for individuals during the execution of tasks. Persons first selectively pay attention to the visual prompt before the target appears (Posner, 1980), then continuously pay attention to the visual prompt while preparing for the appearance of subsequent target (Hoonakker et al., 2017), and finally selectively pay attention to the target when it appears. Appropriate visual prompts can guide people’s attention purposefully (Lukashova-Sanz et al., 2021). Therefore, how to effectively design the visual prompt preceding the appearance of the target in goal-directed tasks, aiming to guide human attention optimally? This will enable individuals to be fully prepared for movement and consequently enhance their performance in accomplishing goal-directed tasks.

At present, the cue-target paradigm is usually used in the research about the effect of visual prompt on attention (Posner, 1980), and the visual prompt is mostly presented in a static form. As the target will appear at different positions in the field of vision during the goal-directed task, the spotlight theory of attention believes that attention, like a spotlight, can focus on a certain area of visual space (Brefczynski and DeYoe, 1999), and arrow cues could guide participants’ attention to focus on the position of the target through behavioral analysis (Zhang et al., 2023). Eimer studied and measured the continuous negative variation (CNV) and the lateralized readiness potential (LRP) triggered by arrow cues, and found that the central arrow cues not only prompted the position information of subsequent targets, but also triggered the process related to motor preparation (Eimer, 1993). Engell used functional magnetic resonance imaging technology to analyze the brain imaging results and found that arrow cues can regulate the activity of ventral frontal parietal attention network (Engell et al., 2010). Despite the widespread use of arrow cues in visual prompts, their static nature may fail to consistently capture human attention during prolonged engagement in goal-directed tasks.

At the same time, some studies believed that some dynamic properties of things, such as the movement or brightness changes of objects, may be the most effective object features that attract human attention in vision (Hillstrom and Chai, 2006). Attention will be attracted by all sudden changes in the visual scene (Miller, 1989). In the study about fixation cues, Zhang believed that the dynamic fixation cue can more effectively guide attention (Zhang et al., 2019). Therefore, in goal-directed tasks, the dynamic preparation prompt is expected to attract human’s attention and make humans prepare for the appearance of targets in advance. However, some studies have shown that the dynamic changes are not necessarily easier to be perceived than static ones (Tversky et al., 2002). In tasks related to learning and memory, for individuals who were difficult to concentrate, the effect of static prompt was better than that of dynamic prompt (Ilgaz et al., 2014). Moreover, even if persons focus on the target to produce a rapid response before the target appears, it may inhibit the execution control to the target (Callejas et al., 2005).

Based on this, considering the variability of the target’s position within the visual field during goal-directed tasks, we used the static central arrow as the cue prompt to indicate the target location. Additionally, taking into account, the widespread use of countdown preparation animations in various motor preparation domains, we utilized the dynamic countdown animation as the preparation prompt. Furthermore, we designed the combination prompt that presented both the static arrow and dynamic countdown simultaneously. In order to explore how to design the visual prompt effectively for goal-directed tasks, the focus of our research is on the guiding effect of different types of visual prompts on attention. We hypothesized that the participant’s brain would show different attention states under different types of visual prompts. Dynamic visual prompts, such as preparation prompts and combination prompts including animation preparation, may be better for the participant’s attention guidance. By comprehensively analyzing the behavior and neurophysiological characteristics of the participants in the task, we proposed a design for visual prompt that effectively guides the attention state of the brain, providing neurophysiological and behavioral foundations for the design of visual prompts in the goal-directed task.

## 2 Methods

### 2.1 Participants

Twenty healthy participants participated in this experiment. All participants were right handed and had normal or corrected-to-normal vision. They were from the Cixi Institute of Biomedical Engineering. Two participants were excluded due to excessive eye movements and too many artifacts on the electroencephalographic (EEG) signals. As a result, the EEG data of 18 participants (11 males and seven females; mean age of 24.8 ± 2.3 years) have been retained. We conducted all experimental procedures according to the Declaration of Helsinki, and before the experiment, all participants signed a written informed consent approved by the local Research Ethics Committee.

### 2.2 Experimental procedure

The experiment was conducted in an electromagnetic shielding room to reduce disturbance from the environment. Before the experiment began, the participants were asked about their emotional and physical condition. All stimuli were on the 23 in display (1,920 × 1,080 pixels, 60 Hz refresh rate), with a viewing distance of 70 cm. They were required to focus on the prompts on the screen to complete the correct response to the target. All visual stimuli appeared on a gray background. They included a white cross (size: 3.52° × 3.52°of visual angle), a white clock (size: 9.73° × 9.73°of visual angle), a white arrow (size: 4.90° × 3.52°of visual angle), and a white pentagon or hexagon (size: 4.90° × 4.90°of visual angle) that appeared at the top or bottom. Participants were asked to use the middle finger of their right hand for key responses, and to avoid any other physical movement except the middle finger movement. They were asked to avoid blinking and swallowing as much as possible. The experiment consisted of five consecutive blocks, and each block contained 80 trials. Each participant had a round of practice before the experiment, which was used to help the participants get familiar with the task rules and key specifications. After each round, the participants could rest, and the rest time was determined by the participants themselves based on their physical condition. After each round of the experiment, the participants were asked how much attention they paid to the experiment and how well they completed it, and whether they were distracted by other stimuli around them.

**Figure 1:**
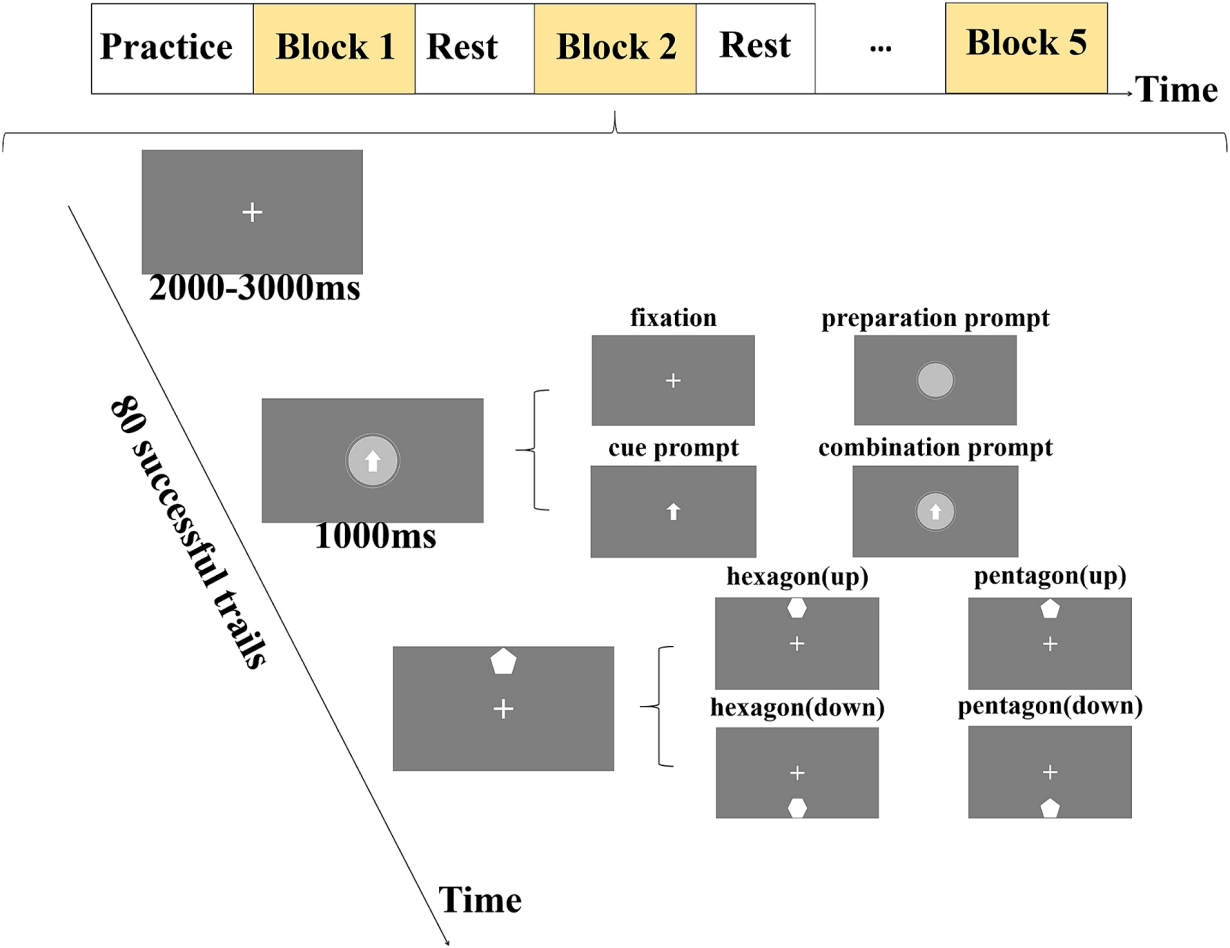
A schematic diagram of experimental design for each experiment. Each experiment consists of three stages. The first stage was the fixation stage (2,000–3,000 ms), in which a white fixation point was presented to remind participants that they could relax appropriately. The second stage was the prompt stage (1,000 ms), in which the visual prompt was presented to remind participants to pay attention to the stimulus and make motor preparation. The third stage was the target stage, where the target was presented and the participants were reminded to perform the key-press response as required.

The experimental example is shown in Figure 1. Each experiment consisted of three stages. The first stage was the fixation stage, and the screen presented the fixation point. The second stage and the third stage took the time when the target appeared as the dividing point, which were the prompt stage and the target stage, respectively. Each experiment started with the appearance of the fixation point, and after a variable interval of 2,000 ms + X (M_x_ = 1,000 ms), the fixation point (no prompt) or visual prompt was randomly presented for 1,000 ms. Among them, the visual prompt included preparation prompt, cue prompt, or combination prompt of cue and preparation information (combination prompt). The preparation prompt was a clock that rotated clockwise and gradually disappeared. The cue prompt was a central arrow, indicating that the target would appear at the top or bottom. The combination prompt was that the clock and arrow appeared simultaneously and gradually disappeared by rotating clockwise. The four types of visual prompts (the cue prompt, the preparation prompt, the combination prompt, and the no prompt) are randomly presented, and each prompt was presented 100 times in each participant’s experiment. Then the target was presented immediately in the target stage. The target included pentagon or hexagon, which appeared at the top or bottom randomly. The target position was the same as the cue position. The number of pentagons and hexagons was 7:3, and the participants were asked to respond only to hexagons by keyboard control. The experiment required the participants to keep their middle finger on the number 5 of the keyboard during the fixation and prompt stages. In the target stage, if the hexagons appeared at the top, the participants were required to press the number 8, and if the hexagons appeared at the bottom, they were required to press the number 2. The experiment required the participants to press the key as soon as possible after the target was presented, and the target disappeared immediately after the key was pressed. If the participants did not press the key, the target would disappear after 1,500 ms.

### 2.3 Data acquisition

The EEG data were recorded from Ag/AgCl electrodes using Neuroscan EEG system. These electrodes were mounted in a 64-channel electrode cap. The electrodes were arranged according to the international 10–20 system. The other two monopolar electrodes were located at the left and right mastoids (M1, M2). Two bipolar electrode pairs were used for the vertical and horizontal electromyography (EOG) recording to detect eye-movements and blinks. The reference electrode (REF) was set on the top of the head, and the ground electrode (GND) on the forehead (AFZ). The 64-channel EEG data we collected covered the whole head. The signal was amplified by SynAmps-2 amplifier with a 0.05–200 Hz bandpass filter and 50 Hz notch filter, and the sampling rate was 1,000 Hz. The impedance between the electrode and the scalp was less than 10 kΩ.

### 2.4 Data analysis

The EEG data were preprocessed offline using EEGLAB (Delorme and Makeig, 2004). The raw data were filtered with a band-pass filter over the range of 0.1–30 Hz and a notch filter at 50 Hz. All EEG signals were then re-referenced to the average of the left and right mastoids. The EEG signals in both the prompt phase and the target phase were divided into time periods of 1,100 ms. The EEG data from −100 ms to 1,000 ms of all stimuli were intercepted, and the baseline correction was performed using −100 ms to 0 ms epoch as a baseline. Eye movements and motion artifacts were removed by the Independent Component Analysis (ICA), and the trials with voltages in the range ± 100 μV were retained. We have calculated the mean and standard deviation of the trials used by all participants for data analysis. In the prompt stage, the final numbers of trials submitted to analysis per participant were 87.11 ± 12.22 (mean ± SD) for the combination prompt, 86.67 ± 11.46 (mean ± SD) for the preparation prompt, 87.78 ± 11.79 (mean ± SD) for the cue prompt, and 88.22 ± 6.63 (mean ± SD) for the no prompt. In the target stage, the final numbers of trials submitted to analysis per participant were 27.67 ± 2.68 (mean ± SD) for the combination prompt, 28.17 ± 3.15 (mean ± SD) for the preparation prompt, 26.22 ± 1.96 (mean ± SD) for the cue prompt, and 25.28 ± 3.08 (mean ± SD) for the no prompt, respectively.

In order to analyze the effects of the cue prompt, the preparation prompt, and the combination prompt on participants’ attention, the EEG responses under different prompts were superimposed and averaged, respectively. At the prompt stage, the whole brain 64 lead analysis showed that the electrophysiological differences among the three prompts were mainly located in the frontal, central, and parietal regions of the brain. Therefore, we selected the average amplitudes of FZ, CZ, and PZ electrodes in a specific time window for analysis. By observing the EEG and brain topographic map after superposition average, we defined the CNV amplitude as the average amplitude of FZ, CZ, and PZ in the period of 600–1,000 ms. In the target stage, the P300 amplitude was calculated as the average voltage in the 350–550 ms time period of the centro-parietal region of interest (ROI) composed of C1, CZ, C2, CP1, CPZ, and CP2 (Luck et al., 2000) when the hexagon appeared.

In this study, the weighted Phase-Lag Index (wPLI) (Vinck et al., 2011) was used to construct the brain functional network. Calculation generated 62 × 62 weighted adjacency matrices, where each value represents the wPLI value between a pair of electrodes. The mathematical expression for calculating wPLI is as follows:


$${\mathrm{wPLI}}_{XY}=\frac{\sum_{t=1}^{n}\left|\mathrm{imag}\left(S_{XYt}\right)\right|\mathrm{sgn}\left(\mathrm{imag}\left(S_{XYt}\right)\right)}{\sum_{t=1}^{n}\left|\mathrm{imag}\left(S_{XYt}\right)\right|}$$

where imag(·) is the imaginary part taken, sgn(·) is the sign function, and $S_{XYt}$ is the cross-spectrum of the variables X(t) and Y(t).

Graph theory was used to quantitatively analyze the guiding effect of different visual prompts on attention. In the quantitative analysis of brain function network, network thresholding is very important. In this study, the density method was adopted as the thresholding strategy. We set the percentage of the density range in the network between 11 and 50%, with a step size of 1%, to obtain the graph theory metric values for each density. We used the Brain Connectivity Toolbox (BCT) (Rubinov and Sporns, 2010) to calculate the clustering coefficient and the characteristic path length in the prompt stage of 600–1,000 ms at each density. For each prompt condition, we calculated the area under the curve (AUC) of these graph theory indicators, which can eliminate the impact of weak connections on quantitative analysis of network topology and reduce bias in brain network functional connectivity analysis.

### 2.5 Statistical analysis

The behavioral and EEG data were compared using the program for statistical analysis (SPSS version 22). Specifically, repeated measures ANOVA was used to analyze the behavioral data, the event-related potentials (ERP) in the target stage, and the AUC results of graph theory indicator for different prompts (the cue prompt, the preparation prompt, the combination prompt, and the no prompt). The ERP of different prompts (the cue prompt, the preparation prompt, the combination prompt, and the no prompt) and different electrode positions (FZ, CZ, and PZ) during the prompt stage were used 3 × 3 repeated measurement ANOVA. In all analyses, a *p* value of <0.05 was considered to represent statistical significance.

## 3 Results

### 3.1 Prompt stage

#### 3.1.1 ERP results

**Figure 2:**
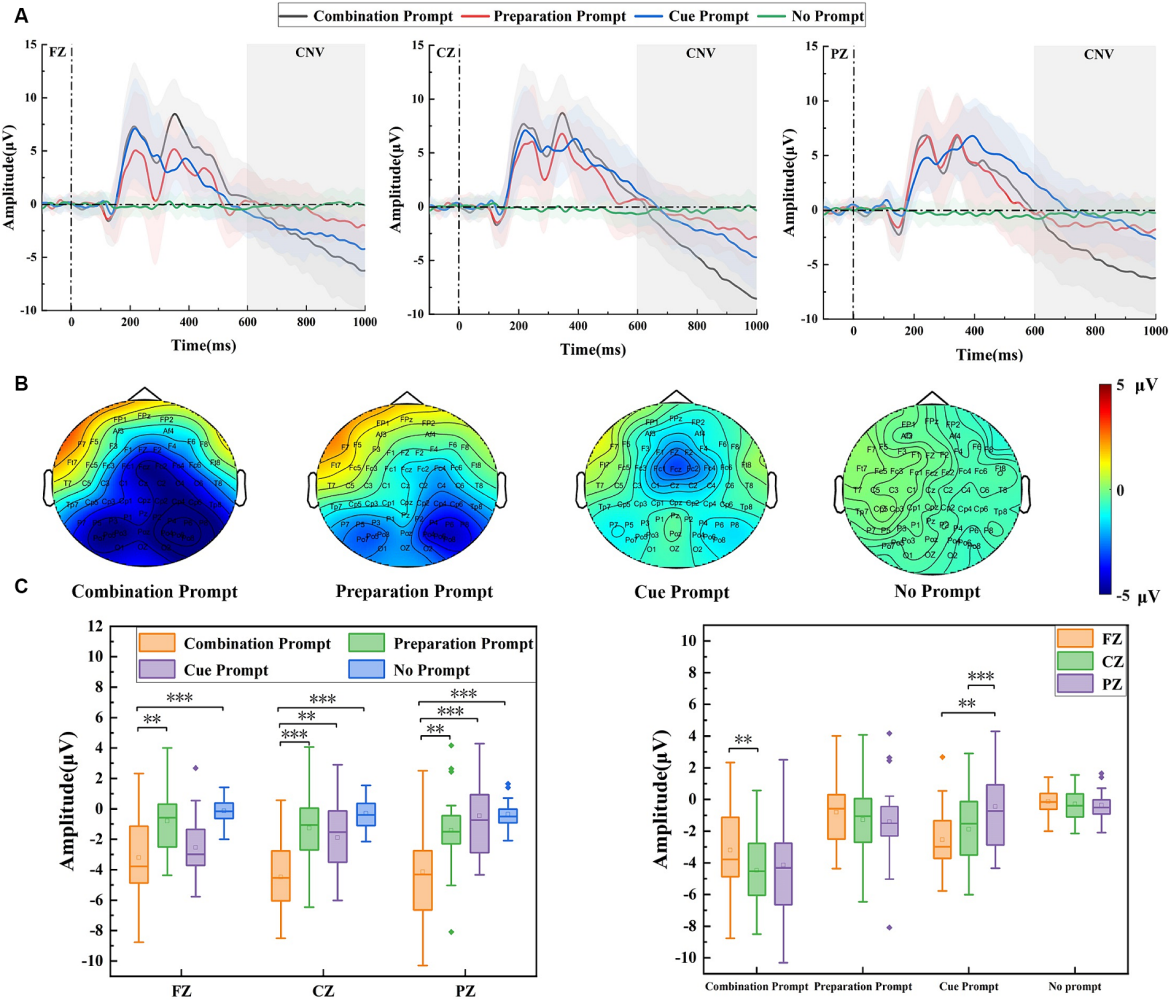
The amplitude differences of brain regions recorded under the combination prompt, preparation prompt, cue prompt, and no prompt. **(A)** ERP waveforms (the average values of all participants) on FZ, CZ, and PZ electrodes under different prompts. Among them, the black line, red line, blue line, and green line represent four prompts: the combination prompt, preparation prompt, cue prompt, and no prompt, respectively. The shaded areas are error bands. **(B)** Brain topographic maps under different prompts, from left to right, is: the combination prompt, preparation prompt, cue prompt, and no prompt. **(C)** The statistical results of CNV amplitude under different prompts, with four different colors representing four different prompt types, a horizontal line representing the median, a small hollow rectangle representing the average, and a solid diamond representing outliers off center (^*^p < 0.05, ^**^p ≤ 0.01, ^***^p ≤ 0.001).

Figure 2A shows the potential waveforms under different prompts on the FZ, CZ, and PZ electrodes at the prompt stage. CNV can be seen after 600 ms under the three prompts. The CNV amplitude is generally considered to be positively correlated with sustained attention (Hasler et al., 2016). From the three waveforms, it can be seen that the CNV amplitude was the largest under the combination prompt on each electrode, indicating that the brain was in a better state of sustained attention under such prompts. Figure 2B shows the brain topographic map from 600 ms to 1,000 ms in the prompt stage. It can be seen from the map that there were differences in the distribution of CNV in the brain regions under the three prompts. The amplitude in the central region of the brain was the largest under the combination prompt, the amplitude in the frontal region of the brain was the largest under the cue prompt, and the amplitude in the partial region of the brain was the largest under the preparation prompt. For different types of prompts, the combination prompt have the highest amplitudes in the frontal, central, and parietal regions of the brain. The cue prompt have greater amplitudes in the frontal and central regions of the brain than the preparation prompt, while the preparation prompt have greater amplitudes in the parietal region of the brain than the cue prompt. Repeated measurement ANOVA was used to analyze the CNV amplitude under different prompt types (the cue prompt, the preparation prompt, the combination prompt, and the no prompt) and different electrodes (FZ, CZ, and PZ), their interaction was significant (*F* = 5.074, *p* = 0.001, η^2^ = 0.183). The main effect of prompt type was significant (*F* = 11.932, *p* < 0.001, η^2^ = 0.345), and the main effect of electrode position was not significant (*F* = 1.721, *p* = 0.193, η^2^ = 0.025). In the simple effect analysis of prompt type, the CNV amplitude was significantly different on FZ electrode (*F* = 8.447, *p* < 0.001, η^2^ = 0.271), CZ electrode (*F* = 13.859, p < 0.001, η^2^ = 0.379), and PZ electrode (*F* = 9.125, *p* < 0.001, η^2^ = 0.287). On FZ electrode, there was a significant difference (*p* = 0.006) in the CNV amplitude between the combination prompt and the preparation prompt, while there was a significant difference (*p* < 0.001) between the combination prompt and the no prompt. On CZ electrode, there was a significant difference (*p* < 0.001) in the CNV amplitude between the combination prompt and the preparation prompt, there was a significant difference (*p* = 0.002) between the combination prompt and the cue prompt, and there was a significant difference (*p* < 0.001) between the combination prompt and the no prompt. On PZ electrode, there was a significant difference (*p* = 0.009) in CNV amplitude between the combination prompt and the preparation prompt, there was a significant difference (*p* < 0.001) between the combination prompt and the cue prompt, and there was a significant difference (*p* < 0.001) between the combination prompt and the no prompt. In the simple effect analysis of electrode position, the CNV amplitude was significant under both the combination prompt (*F* = 9.598, *p* < 0.001, η^2^ = 0.223) and the cue prompt (*F* = 11.221, *p* < 0.001, η^2^ = 0.251), while the CNV amplitude was not significant under both the preparation prompt (*F* = 0.721, *p* = 0.490, η^2^ = 0.021) and the no prompt (*F* = 0.091, *p* < 0.913, η^2^ = 0.003). Among them, under the combination prompt, there was a significant difference (*p* = 0.005) in the CNV amplitude between the FZ electrode and the CZ electrode. Under the cue prompt, there was a significant difference (*p* = 0.003) in the CNV amplitude between the FZ electrode and the PZ electrode, while there was a significant difference (*p* < 0.001) between the CZ electrode and the PZ electrode. The statistical results are shown in Figure 2C.

#### 3.1.2 Graph theoretical analysis

**Figure 3:**
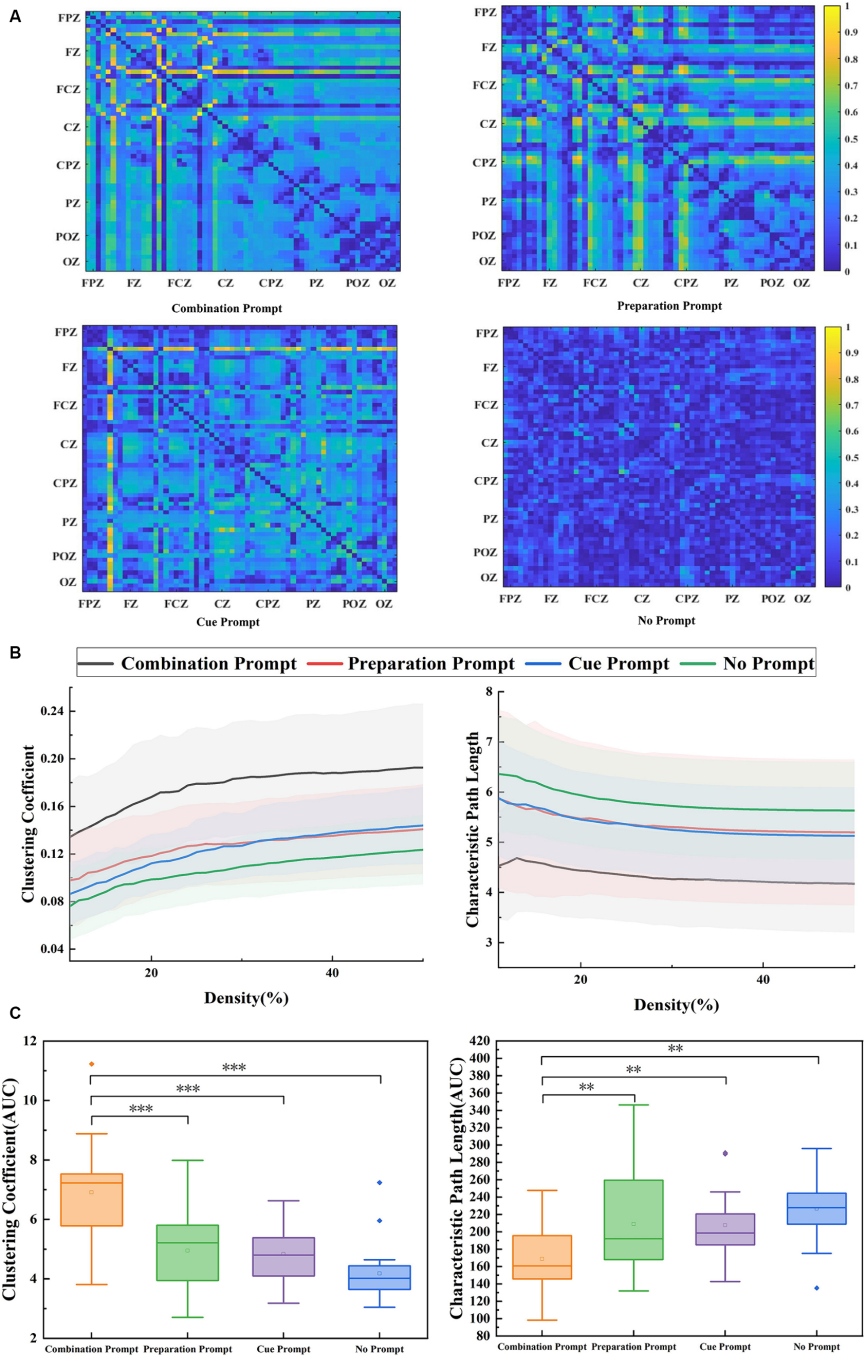
The results of brain functional connectivity analysis recorded under the combination prompt, preparation prompt, cue prompt, and no prompt. **(A)** The brain functional connectivity matrix under different prompts is in the order of the combination prompt, preparation prompt, cue prompt, and no prompt. **(B)** The clustering coefficient and characteristic path length analysis results of brain graph theory indicators (the average values of all participants) under different prompts, where the black line, red line, blue line, and green line represent four prompts: the combination prompt, preparation prompt, cue prompt, and no prompt, respectively. The shaded areas are error bands. **(C)** The statistical results of brain graph theory index AUC under different prompts. The horizontal line represents the median, and the small hollow rectangle represents the average. The *p* value represents the level of significance (^*^p < 0.05, ^**^p < 0.01, ^***^p ≤ 0.001).

Figure 3A shows the adjacency matrix diagram of brain functional connectivity under four conditions, where the whole brain connectivity was closer under the combination prompt, especially in the frontal region related to sustained attention. Figure 3B shows clustering coefficient and characteristic path length of the participants’ brain functional network under four different prompts. Clustering coefficient and characteristic path length can reflect the attention state of the brain (Li et al., 2011). Clustering coefficient of the participants’ brain under the combination prompt was high and characteristic path length was low, indicating that the participants’ attention was more concentrated at this time. As shown in Figure 3C, repeated measure ANOVA was performed on the AUC composed of clustering coefficient (*F* = 16.851, *p* < 0.001) and characteristic path length (*F* = 5.947, *p* = 0.005), and the results showed significant differences. The AUC value composed of clustering coefficient under the combination prompt was significantly higher than that of the cue prompt (*p* < 0.001), the preparation prompt (*p* < 0.001), and the no prompt (*p* < 0.001), while the AUC value composed of characteristic path length under the combination prompt was significantly lower than that of the cue prompt (*p* = 0.005), the preparation prompt (*p* = 0.014), and the no prompt (*p* = 0.008).

### 3.2 Target stage

#### 3.2.1 Behavioral results

In the experiment, the presentation time of the target stimulus was 1,500 ms. Therefore, reactions exceeding 1,500 ms were considered as outliers and excluded. Repeated measure ANOVA was used to analyze the response time and the response accuracy of participants under different types of prompts. The response time of participants to the four different prompts was statistically significant (*F* = 24.428, *p* < 0.001). The response time under the combination prompt (Mean ± SD = 634 ± 150 ms) being significantly faster (*p* < 0.001) than that under the preparation prompt (Mean ± SD = 786 ± 158 ms), it was significantly faster (*p* = 0.002) than that under the cue prompt (Mean ± SD = 762 ± 151 ms), and it was significantly faster (*p* < 0.001) than that under the no prompt (Mean ± SD = 826 ± 150 ms). The response time under the cue prompt was significantly faster (*p* = 0.013) than that under the preparation prompt, and it was significantly faster (*p* = 0.001) than that under the no prompt. The response time under the preparation prompt was significantly faster (*p* = 0.040) than that under the no prompt. However, the accuracy of participants’ responses was almost perfect under the combination prompt (Mean ± SD = 0.996 ± 0.011), the cue prompt (Mean ± SD = 0.998 ± 0.008), the preparation prompt (Mean ± SD = 0.998 ± 0.008), and the no prompt (Mean ± SD = 0.993 ± 0.014), so they cannot be distinguished (*F* = 1.545, *p* = 0.227).

#### 3.2.2 ERP results

**Figure 4:**
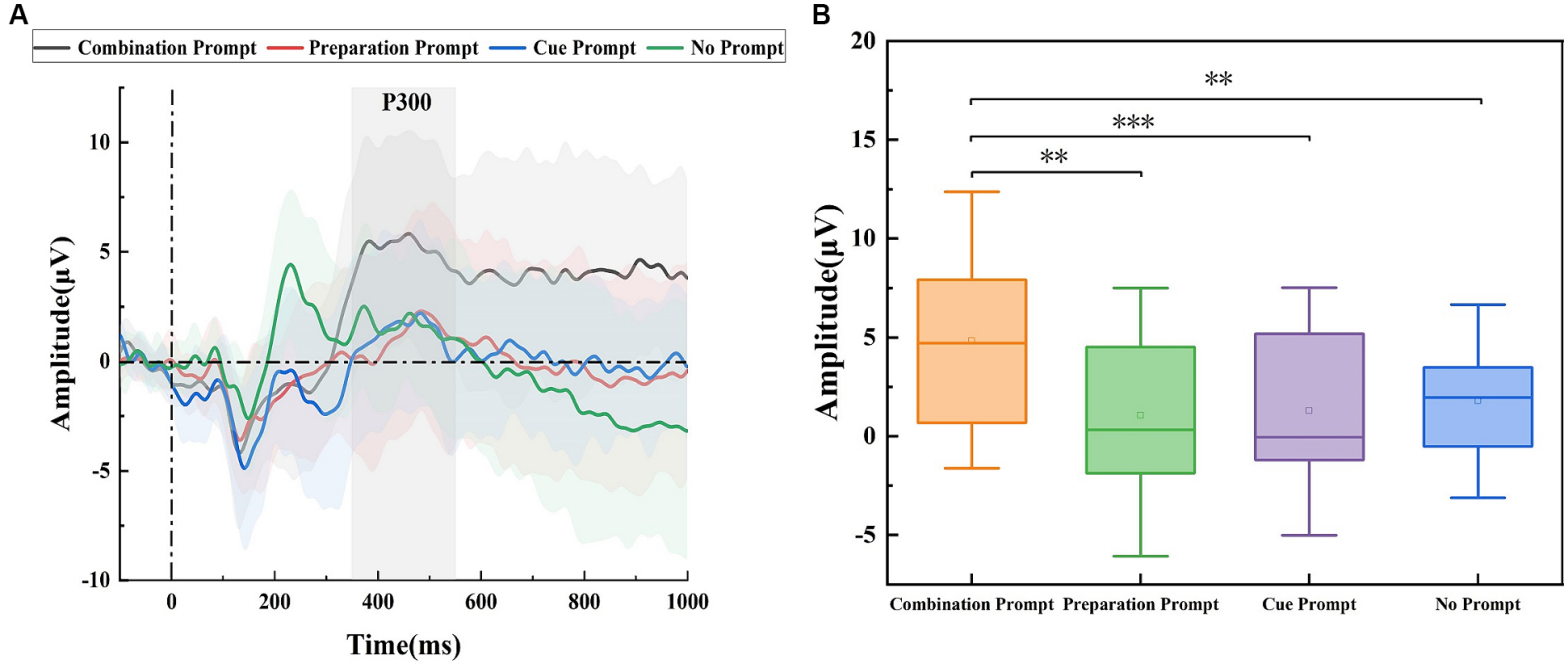
The amplitude differences of brain regions recorded under the combination prompt, preparation prompt, cue prompt, and no prompt. **(A)** ERP waveforms (the average values of all participants) on ROI region under different prompts. Among them, the black line, blue line, red line, and green line represent four prompts: the combination prompt, preparation prompt, cue prompt, and no prompt, respectively. The shaded areas are error bands. **(B)** The statistical results of P300 amplitude under different prompts indicate four different types of prompts, with horizontal lines representing the median and small hollow rectangles representing the average. The *p* value represents the level of significance (^*^p < 0.05, ^**^p < 0.01, and ^***^p ≤ 0.001).

Figure 4A shows the potential waveforms of the target stage. P300 can be seen in the ROI area at 350 ms. P300 is used to measure the participants’ executive control ability and is considered to reflect the brain’s selective attention state (Catalano et al., 2021). The P300 amplitude was the largest under the combination prompt, indicating that the participants’ executive control ability was stronger and their brains’ selective attention state was better under this prompt. The statistical results are shown in Figure 4B. Repeated measure ANOVA was performed on the P300 amplitude of participants under four different prompts, and the results showed significant differences (*F* = 8.929, *p* < 0.001). The P300 amplitude under the combination prompt was significantly higher (*p* = 0.001) than that under the cue prompt, it was significantly higher (*p* = 0.003) than that under the preparation prompt, and it was significantly higher (*p* = 0.037) than that under the no prompt. There was no significant difference (*p* = 1.000) in P300 amplitude between the cue prompt and the preparation prompt, there was no significant difference (*p* = 1.000) in P300 amplitude between the cue prompt and the no prompt, and there was no significant difference (*p* = 1.000) in P300 amplitude between the preparation prompt and the no prompt.

## 4 Discussion

In goal-directed tasks, the visual prompts before the appearance of the target may make the participants ready in advance. This process will be beneficial to their movement (Ju et al., 2018). According to the characteristics of target position changes and the daily application of countdown in motor preparation, we designed three different types of visual prompts: the preparation prompt, the cue prompt, and the combination prompt of cue and preparation information. By recording brain activity, we explored the guiding effect of different types of visual prompts on attention in tasks, and provided the neurophysiological and behavioral foundation for the design of goal-directed tasks. For this reason, after the whole brain 64 lead analysis, we compared the CNV amplitudes of the frontal, central, and parietal regions in different types of visual prompts, the graph theory index clustering coefficient and characteristic path length of brain functional connectivity in the prompt stage, the response time and the P300 amplitude in the central parietal region in the target stage.

Our study found that in the prompt stage, the CNV amplitude of the frontal, central, and parietal regions under the combination prompt was greater than that under the cue prompt and the preparation prompt. We believe that this may be due to the fact that the participants’ attention is more concentrated under the combination prompt than that of cue prompt and preparation prompt, and more attention resources are required to complete the task. Previous literature studies also believe that CNV amplitude reflects the sustained attention necessary to transform sensory information into goal-directed action, and groups that are difficult to concentrate usually show a decrease in CNV amplitude (Mayer et al., 2016). In addition, the brain topographic map results of this study showed that there were differences in the brain activation regions of participants under different types of visual prompts. The CNV amplitude in the frontal region was greater under the cue prompt, the CNV amplitude in the parietal region was greater under the preparation prompt, and the CNV amplitude in the central region was greater under the combination prompt. We believe that this may reflect the different ways in which the brain processes different information. Under the cue prompt, the central arrow more guides the participants to pay attention to the information of the cue itself, which involves the activation of the anterior cingulate, supplementary motor area (SMA), and prefrontal cortex, reflecting the participants’ orienting response to the cue stimulus and early motor preparation (Bender et al., 2007). These areas are usually activated more obviously in the early stage of CNV. However, under the preparation prompt and the combination prompt, CNV exhibited a more pronounced distribution within the central parietal region, predominantly during its later stage. It involves the activation of the pre/primary motor cortex, SMA, posterior parietal and secondary sensory cortex, reflecting the continuous attention state related to the process of brain advanced motor preparation (Bender et al., 2005). It shows that in the goal-directed task with visual prompts, when the participants’ brain processes the visual prompt, they will first notice the cue and process it, and then make preparations to deal with the subsequent target stimulus. For participants with normal cognitive function, the combination prompt of a static arrow and dynamic countdown enhances their sustained attention and facilitates task completion by providing comprehensive preparation. However, this combination prompt will make participants consume more attention resources in the prompt stage. For participants with cognitive impairment, considering their limited attention resources, single cue prompt or preparation prompt can also play a certain role in guiding participants’ attention and helping them make motor preparation when they consume less attention resources. Therefore, the cue prompt or the preparation prompt may be used to guide their attention in goal-directed tasks.

In this study, for participants with normal cognitive function, it can be seen from the brain function connection matrix that the frontal central region of the brain was more closely connected under the combination prompt, and the central parietal lobe of the brain is more closely connected under the preparation prompt. Previous studies believe that the frontal parietal network plays an important role in motor preparation and attention control (Brass and von Cramon, 2002), in which sustained attention is related to the activation of the frontal lobe region, and the central parietal lobe region controls the movement of the human body. We believe it shows that under the guidance of directional cues, participants pay more attention to the information of arrow cues themselves. At this time, the participation of attention makes the brain have specific cognition and planning for subsequent targets, while without the guidance of directional information, participants only make simple motor preparation for subsequent targets. At the same time, under the combination prompts of both the preparation animation and the cue arrow, the participants’ attention related brain regions were activated more than the single cue prompt, indicating that the brain needs to have the process of information processing for the motor preparation with attention participation. Under the interaction of attention and motor preparation, the participants’ motor performance at the target stage was the best under the combination prompt. In graph theory analysis, clustering coefficient is used to measure the separation characteristics of the brain network topology. It can describe the tightness of the connection between the neighbor nodes of a node and can well reflect the local aggregation of the brain network (Lehrer, 2009). The increases in clustering coefficient under the combination prompt indicated that the connection strength between the neighbor nodes in the brain network was improved, and the processing speed of local information was improved. Characteristic path length is used to measure the minimum path between nodes, which can reflect the global information transmission ability of the brain network, and also can represent the functional integration ability of the brain network (Achard and Bullmore, 2007; Sun et al., 2014). Under the combination prompt, the brain showed low characteristic path length, which indicated that the brain network was more efficient in transmitting information. Previous studies have shown that the brain of participants in the state of sustained attention usually shows the characteristics of high clustering coefficient and low characteristic path length (Dimitrakopoulos et al., 2018; Fisher, 2019), indicating that the brain has better sustained attention under the combination prompt. At the same time, in the target stage, the behavioral analysis results showed that the participants’ response to the target was faster, and the ERP results showed that the P300 amplitude in the central parietal region was greater. In previous studies, the P300 amplitude in the central parietal region was considered to be related to the ability of executive control to the target, reflecting the participants’ selective attention. When the P300 amplitude of the participant was small, it showed that the cognitive function of the participant was at a poor level (Hermens et al., 2010). When the preparation and cue were combined, the P300 amplitude of the participant was larger, indicating that the participants’ ability of executive control to the target was stronger. We believe that under the combination prompt, which is a dynamic prompt containing arrow information; participants pay more attention, consume more attention resources, and have higher brain processing efficiency. At the same time, better sustained attention in the prompt stage helps participants have stronger executive control ability and better behavioral performance in the target stage.

## 5 Conclusion

In general, in order to explore how we should design visual prompts in goal-directed tasks, our research focuses on the guiding effect of different types of visual prompts on attention, and makes a comprehensive analysis of the participants’ behavior and brain electrophysiological characteristics in the task. The results showed that compared with the cue prompt and the preparation prompt, under the combination prompt of cue and preparation information, the CNV amplitude was larger, clustering coefficient was higher, and characteristic path length was lower during the prompt stage. Additionally, the brain reacts faster, and the P300 amplitude was larger during the target stage. Our results show that for participants with normal cognitive function, the combination prompt can better guide the participants’ continuous attention in the prompt stage, and make the participants well prepared for movement, so that in the target stage, they have better executive control ability to achieve faster response to the target. However, because the combination prompt will make participants consume more attention resources in the prompt stage, for participants with impaired cognitive function, due to their limited attention resources, we believe that single cue prompt or preparation prompt may be used. At present, the rehabilitation effect of goal-directed tasks on stroke patients has been confirmed. This study is only carried out for healthy participants with normal cognitive function. Further research will focus on how to design visual prompts in goal-directed tasks for patients with impaired cognitive function.

## Data availability statement

The original data and materials presented in this article can be obtained from the corresponding authors upon request.

## Ethics statement

The studies involving humans were approved by Ningbo Cixi Institute of Biomedical Engineering. The studies were conducted in accordance with the local legislation and institutional requirements. The participants provided their written informed consent to participate in this study.

## Author contributions

YLi: Conceptualization, Formal analysis, Investigation, Methodology, Software, Visualization, Writing – original draft. YY: Data curation, Formal analysis, Investigation, Methodology, Writing – review & editing. BY: Data curation, Investigation, Writing – review & editing. YLu: Data curation, Software, Writing – original draft. HZ: Conceptualization, Funding acquisition, Methodology, Writing – review & editing. MT: Conceptualization, Methodology, Project administration, Writing – review & editing. GZ: Conceptualization, Funding acquisition, Methodology, Project administration, Supervision, Writing – review & editing. JX: Conceptualization, Funding acquisition, Methodology, Project administration, Resources, Supervision, Writing – review & editing.
